# Canagliflozin Delays Aging of HUVECs Induced by Palmitic Acid via the ROS/p38/JNK Pathway

**DOI:** 10.3390/antiox12040838

**Published:** 2023-03-30

**Authors:** Wenhui Hao, Wenjie Shan, Fang Wan, Jingyi Luo, Yaoyun Niu, Jin Zhou, Yaou Zhang, Naihan Xu, Weidong Xie

**Affiliations:** 1 State Key Laboratory of Chemical Oncogenomics, Shenzhen International Graduate School, Tsinghua University, Shenzhen 518055, China; 2 Shenzhen Key Laboratory of Health Science and Technology, Institute of Biopharmaceutical and Health, Tsinghua University, Shenzhen 518055, China; 3 Open FIESTA Center, Shenzhen International Graduate School, Tsinghua University, Shenzhen 518055, China; 4 Institute for Ocean Engineering, Shenzhen International Graduate School, Tsinghua University, Shenzhen 518055, China

**Keywords:** canagilflozin, aging, p38MAPK, JNK, blood vessel

## Abstract

Vascular aging is an important factor contributing to cardiovascular diseases, such as hypertension and atherosclerosis. Hyperlipidemia or fatty accumulation may play an important role in vascular aging and cardiovascular diseases. Canagliflozin (CAN), a sodium-glucose cotransporter inhibitor, can exert a cardiovascular protection effect that is likely independent of its hypoglycemic activities; however, the exact mechanisms remain undetermined. We hypothesized that CAN might have protective effects on blood vessels by regulating vascular aging induced by hyperlipidemia or fatty accumulation in blood vessel walls. In this study, which was undertaken on the basis of aging and inflammation, we investigated the protective effects and mechanisms of CAN in human umbilical vein endothelial cells induced by palmitic acid. We found that CAN could delay vascular aging, reduce the secretion of the senescence-associated secretory phenotype (SASP) and protect DNA from damage, as well as exerting an effect on the cell cycle of senescent cells. These actions likely occur through the attenuation of the excess reactive oxygen species (ROS) produced in vascular endothelial cells and/or down-regulation of the p38/JNK signaling pathway. In summary, our study revealed a new role for CAN as one of the sodium-dependent glucose transporter 2 inhibitors in delaying lipotoxicity-induced vascular aging by targeting the ROS/p38/JNK pathway, giving new medicinal value to CAN and providing novel therapeutic ideas for delaying vascular aging in patients with dyslipidemia.

## 1. Introduction

Aging is a complex biological process characterized by the gradual deterioration of the structures and functions of multiple organs; vascular remodeling, endothelial dysfunction and loss of vascular function are all important features of aging. Cardiovascular diseases, such as hypertension and atherosclerosis, account for more than 40% of all deaths in patients aged 65–74 years and nearly 60% of deaths in patients aged 85 years or older [1]. The gradual loss of blood vessel function during aging is an important factor contributing to the high incidence of cardiovascular disease in the elderly [2,3]. The vascular aging process plays a role not only in macrovascular diseases (e.g., atherosclerotic diseases of the large arteries [4]) but also in many microvascular pathologies [5]. The vascular aging phenotype can be reversed and vascular aging can be delayed if effective interventional strategies are used [6,7,8]. However, the vascular aging mechanism and the ideal drugs remained undetermined.

Senescence is a cellular program that induces stable growth arrest with marked phenotypic changes, including chromatin remodeling, metabolic reprogramming, increased autophagy and secretion of complex pro-inflammatory factors [9,10]. Endothelial cells constitute one of the most important parts of the blood vessel with direct contact with blood components. Aging endothelial cells may be associated with increased secretion of inflammatory factors and loss of blood vessel functions [11]. Endothelial cell aging may be induced by many factors in the blood. Among those factors, hyperlipidemia or fatty accumulation may have a key role in mediating vascular aging [12]. 

Canagliflozin (CAN) is a novel glucose-lowering drug that is primarily used to treat type 2 diabetes. It is a sodium-dependent glucose transporter 2 (SGLT2) inhibitor that increases urinary glucose excretion by inhibiting such transporters in the S1 segment of the proximal tubule of the kidney, thereby achieving a hypoglycemic effect. Emerging studies suggest that SGLT2 inhibitors, including CAN, have significant cardioprotective effects. Furthermore, more and more studies have confirmed that CAN has new activities independent of hypoglycemic effects [13,14,15]. CAN increases the energy expenditure of adipocytes without SGLT2 inhibition, thereby regulating energy homeostasis [16,17]. In our previous studies, we found that CAN had a cardiovascular protective effect on palmitic acid-induced cardiomyocytes [18,19]. Furthermore, CAN extended the lifespan of male mice by delaying the breakdown of carbohydrates in the body [20]. However, the actual mechanism requires further investigation. 

In this study, we hypothesized that CAN might have a direct effect on delaying vascular aging induced by hyperlipidemia and a protective effect against cardiovascular diseases. We examined the role of CAN in delaying cellular senescence induced by palmitic acid (PA); investigated the regulatory pathways of CAN in relation to senescent cells in terms of the senescence-associated secretory phenotype (SASP), DNA damage and the cell cycle; and determined the mechanism by which CAN regulates cellular senescence through the ROS/p38/JNK pathway.

## 2. Materials and Methods

### 2.1. Molecular Docking Assay 

Firstly, we predicted potential targets of CAN and conducted a pathway enrichment analysis using SwissTargetPrediction (http://www.swisstargetprediction.ch/, accessed on 15 May 2020), employing our previous method [18]. We found that the MAPK signaling pathway was listed as one of the top 20 signaling pathways. p38 MAPK (MAPK14) and JNK (MAPK8) are two key predicted factors responsible for cell stress, inflammation and senescence [21]. Following that, we selected these two interesting factors to conduct a molecular docking assay with CAN using SwissDock, an online sever for small-molecule protein docking [22]. The p38 MAPK and JNK crystal structures were obtained using the Protein Data Bank (PDB ID: 4MYG and 3O2M). Small-molecule structures were obtained from Drugbank (https://go.drugbank.com/, accessed on 3 November 2022); small-molecule protein structures were preprocessed before docking using Moe 2009 software to remove water molecules from the structures and to add local charges to all atoms with hydrogen atoms. The molecular docking model and affinity energy for CAN with p38 MAPK and JNK were analyzed using SwissDock (www.swissdock.ch, accessed on 5 November 2022) and the results were visualized using the software Chimera v1.14 [23].

### 2.2. Cell Culture and Treatment 

Human umbilical vein endothelial cells (HUVECs) were purchased from the Cell Resource Centre of the Shanghai Institute for Biological Science, Chinese Academy of Sciences, Shanghai, China. The cells were maintained at 37 °C in a humidified atmosphere of 95% air and 5% CO_2_. The cell culture medium consisted of Dulbecco’s modified Eagle medium (Gibco, Waltham, MA, USA) with high glucose supplemented with 10% fetal bovine serum (Invitrogen Carlsbad, Carlsbad, CA, USA) and 1% penicillin-streptomycin antibiotic (Gibco™, ThermoFisher Scientific, Waltham, MA, USA). The liquefied PA was gradually diluted with sterilized 5% bovine serum albumin (BSA, Biofrom, Guangzhou, China; g/mL, BSA/PBS) solution to obtain a PA stock solution (62.5 mM), which was stored at −20 °C for further assays. CAN, SB230580, SP100625 and NAC were obtained from MedChemExpress (Monmouth Junction, NJ, USA), dissolved in dimethyl sulfoxide (DMSO; Sangon, Shanghai, China) and stored at −20 °C for further assays. The cells with exponential growth were incubated with 300 µM PA (Sigma-Aldrich, St. Louis, MO, USA) for 24 h to generate the aging model control group. Different concentrations of CAN (2.5, 5 and 10 μg/mL), SB230580 (10 µM), SP100625 (10 µM) and NAC (1 mM) were added to investigate the protective effects on PA-induced HUVECs. The NOR group was treated with the vehicles DMSO and BSA as a control. In each group, the final concentrations of DMSO and BSA were 0.1% (*v*/*v*) and 300 µM, respectively. 

### 2.3. Senescence-Associated β-Galactosidase Staining Assay

An SA β-galactosidase staining kit was purchased from Beyoncé Biotechnology Co (Shanghai, China). The SA β-galactosidase staining kit was used to detect the activity of senescence-specific β-galactosidase. The cells were inoculated in 96-well plates for 24 h. The cell culture was aspirated, washed once with PBS, treated with 100 μL of β-galactosidase staining fixative and fixed for 15 min at room temperature. The 1000 μL cell-staining working solution consisted of 10 μL β-galactosidase staining solution A, 10 μL β-galactosidase staining solution B, 930 μL β-galactosidase staining solution C and 50 μL X-Gal solution. After the cell fixative was aspirated, the cells were washed with PBS thrice for 3 min each time. Then, we aspirated the PBS and added 100 μL of staining working solution to each well. We sealed the 96-well plates using a sealing film and incubated them overnight at 37 °C in a CO_2_-free incubator for observation under a Leica DMI6000 B inverted microscope (Leica Microsystems, Weztlar, Germany). Cell counts were performed using ImageJ software (National Institutes of Health, Bethesda, MD, USA), and quantitative results were obtained by dividing the number of positive cells in the field of view by the total number of cells. 

### 2.4. Cellular ROS Assay—DCFH-DA Probe

Cells were inoculated in 96-well plates for 24 h. A DCFH-DA probe (Beyotime Biotechnology, Shanghai, China) was diluted with serum-free culture medium at 1:1000 to a final concentration of 10 μmol/L. The cell culture medium was removed, the diluted probe was added and the cells were incubated at 37 °C for 20 min in a cell incubator. The medium was aspirated and washed three times with PBS, and the cells were observed and photographed under a fluorescent microscope (Leica Microsystems, Weztlar, Germany).

For ROS quantification experiments, the cells were inoculated in 12-well plates and subjected to the same treatment as above. After incubation was completed, the cells were digested into Eppendorf tubes using trypsin. The average fluorescence intensity of cells was measured using flow cytometry (Beckman, Brea, CA, USA).

### 2.5. Cell Viability—MTT Assay

Cell viability was assayed using MTT (3-(4,5-dimethylthiazol-2-yl)-2,5-diphenyltetrazolium bromide) (Coolaber, Beijing, China). MTT was prepared in sterile PBS as a 5 mg/mL stock solution, fully dissolved and then filtered for sterilization using a 0.22 µm needle filter (PALL, New York, NY, USA). Cells were inoculated in 96-well plates. After 24 h of the treatment, 20 µL of the MTT solution was added to each well and further incubated at 37 °C for 4 h in the cell culture incubator. Then, the cell medium was removed, the formed formazan was dissolved with 200 µL DMSO solution, and the absorbance at 490 nm was analyzed with an Epoch microplate spectrophotometer (Bio-Tek, Winooski, VT, USA).

### 2.6. Western Blot

The cells were inoculated in 6-well plates for 24 h. Then, the culture dishes were placed on ice and washed twice with ice-cold PBS. Cell lysate (Beyotime Biotechnology, Shanghai, China) reagent was added and cell lysis was collected into EP tubes. Then, cell lysis was centrifuged at 12,000× *g* for 10 min at 4 °C and the supernatant was collected. Sample preparation was performed after determination of the supernatant protein concentration with the Bradford assay (Beyotime Biotechnology, Shanghai, China). Subsequently, equal amounts of protein (30 μg/lane) were separated using 12.5% sodium dodecyl sulfate polyacrylamide electrophoresis gel (Epizyme Biotech, Shanghai, China) and then transferred to nitrocellulose membranes (Pall, New York, NY, USA). The nitrocellulose membranes with the transferred proteins were then incubated for 2 h at room temperature using 5% skim milk (Epizyme Biomedical Technology Co., Ltd., Shanghai, China), incubated overnight at 4 °C using primary antibodies and visualized using enhanced luminescence solution (Thermo Fisher Scientific Inc., Waltham, MA, USA) the following day after 2 h incubation using secondary antibodies. The specific primary antibodies used for the Western blot analysis against protein—namely, TNF-α (1:1000), IL-6 (1:1000), HMGB1 (1:1000), matrix metalloproteinase 3 (MMP3) (1:1000), p-H2A.X (1:1000), lamin B1 (1:1000), p53 (1:1000), p21 (1:1000), p16 (1:1000), p38 (1:1000), p-p38 (1:1000), JNK (1:1000), p-JNK (1:1000), and actin (1:1000)—were purchased from Cell Signaling Technology (Boston, MA, USA). Actin (1:50,000) was purchased from Sigma-Aldrich^®^ (Darmstadt, Germany). The secondary antibodies were purchased from Cell Signaling Technology (Boston, MA, USA). Protein strips were quantified using ImageJ software (National Institutes of Health, Bethesda, MD, USA).

### 2.7. Cell Cycle

The cells were inoculated in 6-well plates for 24 h. Then, the cell culture medium was carefully aspirated and the cells were digested with trypsin to prepare a single cell suspension. After centrifugation (1000× *g*, 5 min), the cell pellets were collected. The cell pellets were washed once with ice-cold PBS and collected by centrifugation. The cell pellets were gently mixed with 1 mL of pre-chilled 70% ethanol, stored overnight at 4 °C and then centrifuged (1000× *g*, 5 min) to precipitate the cells, which were then washed once with ice-cold PBS. The staining solution consisted of 0.5 mL staining solution, 10 μL PI solution and 10 μL RNase A solution. Approximately 0.5 mL of the configured staining solution was added to each cell sample. The cells were gently mixed and resuspended, incubated for 30 min at 37 °C, shielded from light and analyzed using a flow cytometer (Beckman Coulter Life Sciences, Brea, CA, USA).

### 2.8. Statistical Analysis

GraphPad Prism 8.0 software (GraphPad Software, San Diego, CA, USA) was used for the statistical analysis. All the experiments were repeated three or more times independently and the data are expressed as means ± standard deviation (SD.). Differences between groups with statistical significance were calculated using ANOVA followed by Tukey’s post hoc test. *p* < 0.05 was considered statistically significant.

## 3. Results and Discussion

### 3.1. Molecular Docking Assay

**Figure 1:**
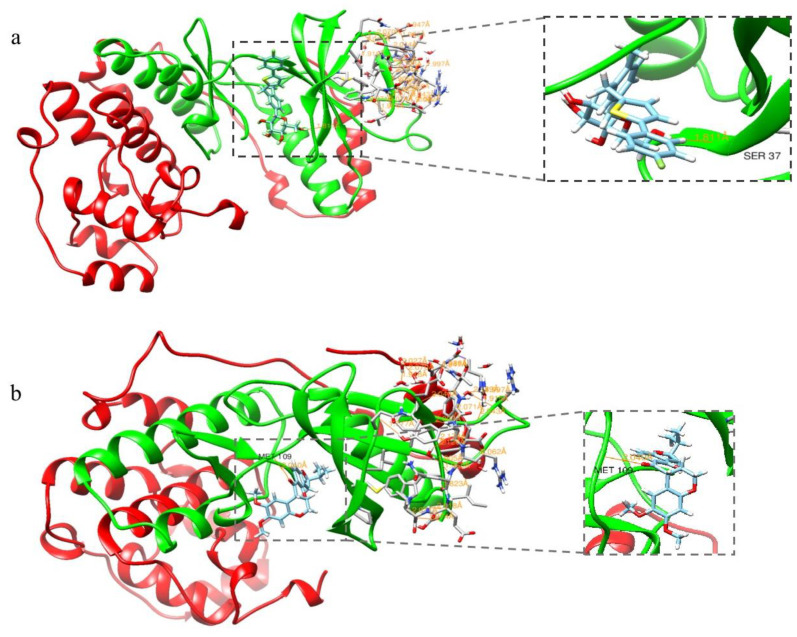
Molecular docking results for p38 with canagliflozin and the positive drug. (**a**) Docking results for p38 and canagliflozin. (**b**) Docking results for p38 and the positive drugs.

**Figure 2:**
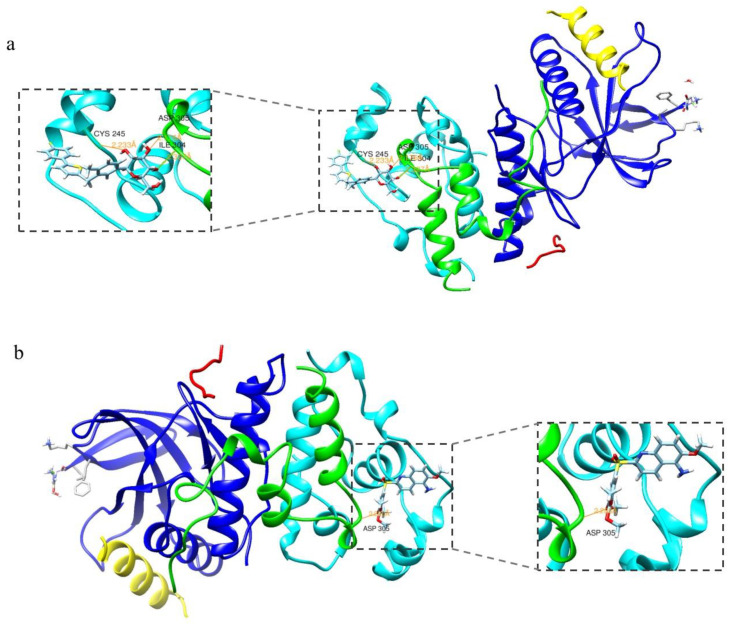
Molecular docking results for JNK with canagliflozin and the positive drug. (**a**) Docking results for JNK and canagliflozin. (**b**) Docking results for JNK and the positive drugs.

Canagliflozin was subjected to molecular docking experiments with p38 protein, and the docking results for the p38-positive drug 3,4-DIHYdroxy-benzenepropanoic acid (p38 metathesis inhibitor) were used as a control analysis. Generally, we believe that ∆G > −4.25 kcal/mol does not represent stable binding between the small-molecule compound ligand and the protein receptor, while ∆G < −7.0 kcal/mol indicates that the two are able to bind stably [24]. The results of the molecular docking calculated in SwissDock software showed that the energy of the binding of canagliflozin to p38 protein was −8.00 kcal/mol, while the energy of the binding of the p38-positive drug to p38 protein was −7.29 kcal/mol, indicating that p38 and Canagliflozin have strong binding abilities and are capable of stable binding, allowing interaction to occur. Figure 1 demonstrates the optimal docking conformation for the binding of the p38-positive drug and canagliflozin to p38 protein. Canagliflozin formed a hydrogen bond with the SER37 residue of p38 protein and the positive drug formed a hydrogen bond with p38 at the MET109 residue, indicating a theoretical interaction between canagliflozin and p38. The binding energy of JNK to canagliflozin was −8.18 kcal/mol, and the binding energy of MPT0B392, the activator of JNK, to JNK was −8.31 kcal/mol, which also indicated that canagliflozin and JNK theoretically interact and are capable of stable binding (Figure 2). The molecular docking assay indicated that p38 and JNK might be the action targets of CAN and provided an important hint for the following experiments.

### 3.2. CAN Delays PA-Induced HUVEC Cell Senescence

**Figure 3:**
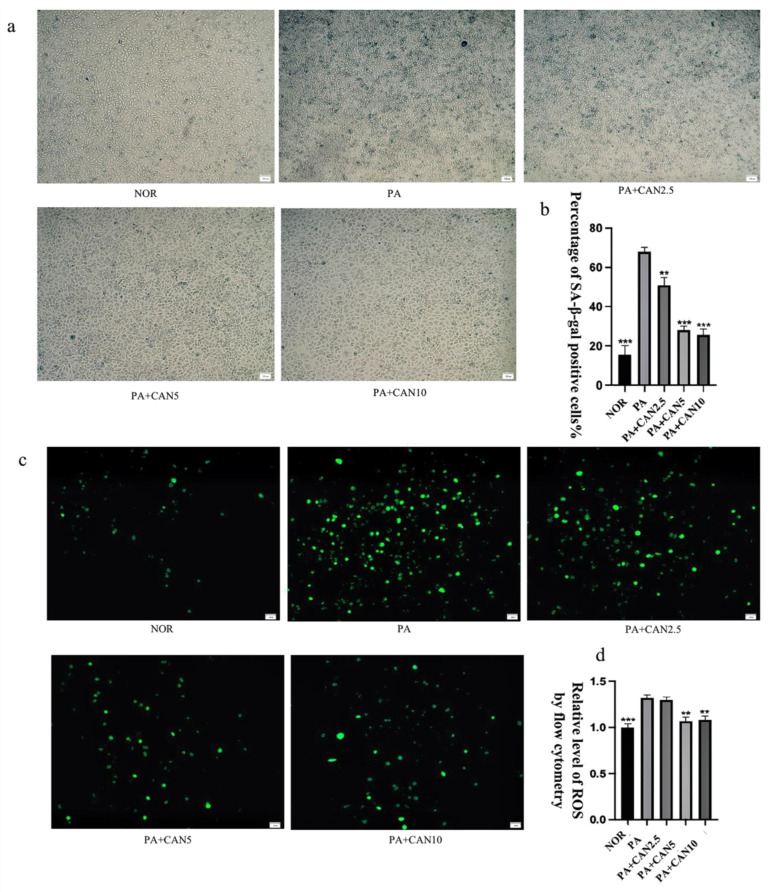
Canagliflozin delays PA-induced aging of HUVECs. (**a**,**b**) CAN reduced the number of β-galactosidase staining-positive cells in a concentration-dependent manner (2.5 μg/mL, 5 μg/mL, 10 μg/mL). (**c**,**d**) CAN reduced the abnormal elevation of intracellular ROS due to senescence and the outcome was concentration-dependent (2.5 μg/mL, 5 μg/mL, 10 μg/mL). (**d**) Results of ROS quantification using flow cytometry. Data are expressed as the means ± SD (*n* = 3); ** *p* < 0.01, *** *p* < 0.001 vs. PA. All images are magnified to 100×.

The drug concentration of CAN was first determined using the MTT method, as shown in Figure S1. Cell viability was above 90% at CAN concentrations below 10 μg/mL, a value which can be considered non-cytotoxic. Thus, 2.5, 5 and 10 μg/mL of CAN were selected. β-galactosidase staining is an important marker for assessing cellular senescence, and senescence-associated (SA) β-gal-positive cells showed an age-dependent increase [25]. Inflammation and oxidative stress are important factors contributing to cellular senescence [26], and the saturated fatty acid PA induces oxidative stress and inflammation [27]. The number of SA β-gal-positive cells significantly increased after treatment with 300 μM PA for 24 h compared to the NOR group. Moreover, the number of positive cells significantly decreased in a concentration-dependent manner after treatment with 2.5, 5 and 10 μg/mL CAN (Figure 3a,b). The well-known free radical theory on aging proposes that high levels of ROS play an important role in the aging process [28] and that an increase in ROS can significantly contribute to the development of human aging and related diseases [29]. As shown in Figure 3c,d, the intracellular ROS content increased significantly after PA treatment, decreased after the addition of CAN treatment and showed CAN concentration dependence. CAN at a concentration of 10 μg/mL minimized the number of SA β-gal-positive cells, as well as the ROS content, but the difference was not significant compared to the results with 5 μg/mL. Therefore, 5 μg/mL of CAN was selected for subsequent testing.

### 3.3. CAN Regulates SASP and Senescence Signature Protein Content

**Figure 4:**
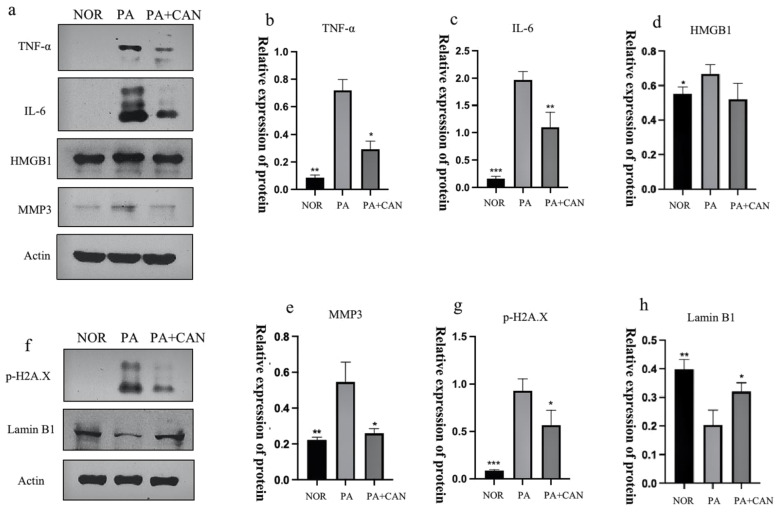
CAN regulated the senescence-associated secretory phenotype (SASP) and senescence signature protein content. (**a**–**e**) CAN (5 μg/mL) inhibited PA-induced increases in TNF-α, IL-6, HMBG1 and MMP3. (**f**–**h**) CAN (5 μg/mL) inhibited PA-induced increases in p-H2A.X and lamin B1 reduction. Data are expressed as the means ± SD (*n* = 3); * *p* < 0.05, ** *p* < 0.01, *** *p* < 0.001 vs. PA.

Senescent cells can produce and secrete a variety of extracellular modulators, including cytokines, chemokines, proteases, growth factors and bioactive lipids, which, in turn, are denoted as the senescence-associated secretory phenotype (SASP) [30]. The SASP can alter the cellular microenvironment and have detrimental influences on neighboring cells [31], so it was essential to explore the changes in the SASP in this model. As shown in Figure 4, expressions of the inflammatory factors TNF-α, IL-6, HMGB1 and MMP3 were low in the NOR group, significantly increased in the PA group and exhibited a significant decrease with the addition of CAN (Figure 4a–e). In addition to the SASP, cell nuclear layer proteins are gradually reduced during cellular senescence, so lamin B1, a cell nuclear layer protein, can be used as a tissue-based biomarker of senescence [32]. The experimental results showed that lamin B1 expression was significantly reduced in the PA group compared to the NOR group, and CAN could significantly improve lamin B1 reduction (Figure 4h). To further verify the ameliorative effect of CAN on DNA damage to senescent cells, we also examined the content of histone p-H2A.X, a marker of DNA damage (Figure 4g). The results showed that CAN interfered with and ameliorated DNA damage caused by PA.

### 3.4. CAN Regulates Cell Cycle Arrest Due to Aging

**Figure 5:**
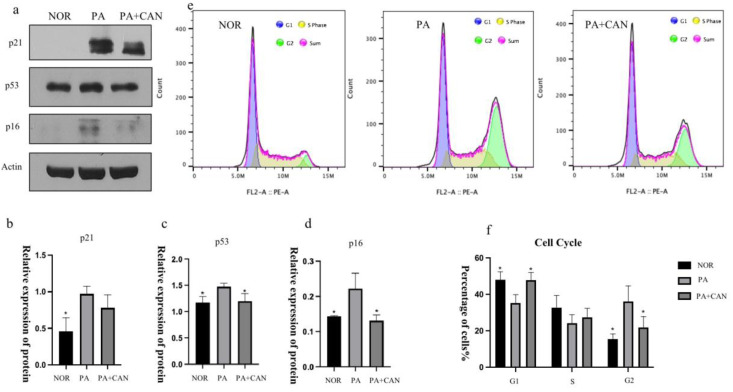
CAN regulated the cell cycle of senescent HUVECs. (**a**–**d**) CAN (5 μg/mL) reduced PA-induced elevation of cell cycle regulators p21, p53 and p16. (**e**,**f**) CAN (5 μg/mL) ameliorated PA-induced cell cycle arrest in HUVECs. Data are expressed as the means ± SD (*n* = 3); * *p* < 0.05 vs. PA.

Another common feature of cellular senescence is cell cycle arrest, a finding that originated when researchers initially observed that human fibroblasts could only undergo division for a limited number of generations in vitro [33]. Mao et al. [34] found that a large number of human fibroblasts with replicative senescence were in a state with 4N DNA content, demonstrating cycle arrest in the G2 phase and an inability to enter mitosis. As shown in Figure 5e–f, cell cycle assays revealed that numerous cells in the PA group were blocked in the G2 phase, and CAN was able to reverse this cell cycle arrest and achieve the same division state as the NOR group. Both p53 and p21 are key transcription factors, and in addition to regulating the cell cycle, activation of p53 and p21 also regulates cellular senescence and organismal aging [35]. p16INK4a, a cyclin-dependent kinase inhibitor, tumor suppressor and biomarker of aging, is another major mediator for cellular senescence. The gradual accumulation of p16 expression during physiological aging and several aging-associated diseases directly implicates this well-established effector of senescence in the aging process [36]. To further explore whether CAN improved cellular senescence by affecting the cell cycle, we examined the expression of p53, p21 and p16 in HUVECs and verified that the levels of the three proteins were significantly higher in the PA group relative to the NOR group. Furthermore, CAN treatment was able to reverse this situation, resulting in a decrease in the expression of cell cycle inhibitors (Figure 5a–d).

### 3.5. CAN Alleviates PA-Induced HUVEC Replicative Senescence by Inhibiting p38 Activation

**Figure 6:**
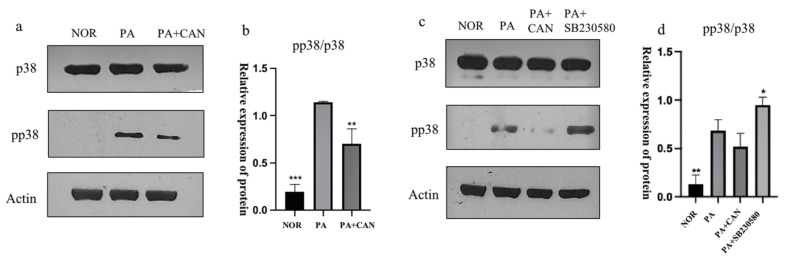
CAN inhibited p38 protein activation. (**a**,**b**) CAN (5 μg/mL) reduced the phosphorylation level of p38 protein. (**c**,**d**) The effect of the p38 inhibitor SB230580 (10 μM) on p38. Data are expressed as the means ± SD (*n* = 3); * *p* < 0.05, ** *p* < 0.01, *** *p* < 0.001 vs. PA.

The p38MAPK pathway is a key regulator of the biosynthesis of pro-inflammatory cytokines that may contribute to chronic low-grade inflammation during aging, and the elevated protein levels of the phosphorylated active form of p38MAPK (phospho-p38 MAPK) with age are tissue-specific [37]. As shown in Figure 6a,b, Western blot analysis of p38 expression levels in HUVECs revealed that the p38 protein was significantly activated in the NOR group and that CAN significantly attenuated the increase in the pp38 (phospho-p38 MAPK)/p38 ratio, thereby indicating that CAN inhibited the activation of the p38 pathway in HUVEC cells. SB230580, a selective inhibitor of p38, had no effect on the activation of intracellular p38. SB203580 effectively inhibited the activity of p38 MAPKs by inhibiting the activation of MAPKAP-K2, a specific physiological substrate of p38MAPK [38]. We treated cells with SB230580 and verified that the inhibitor did not have an effect on the phosphorylation of p38 but had an effect on protein activity (Figure 5c,d). 

**Figure 7:**
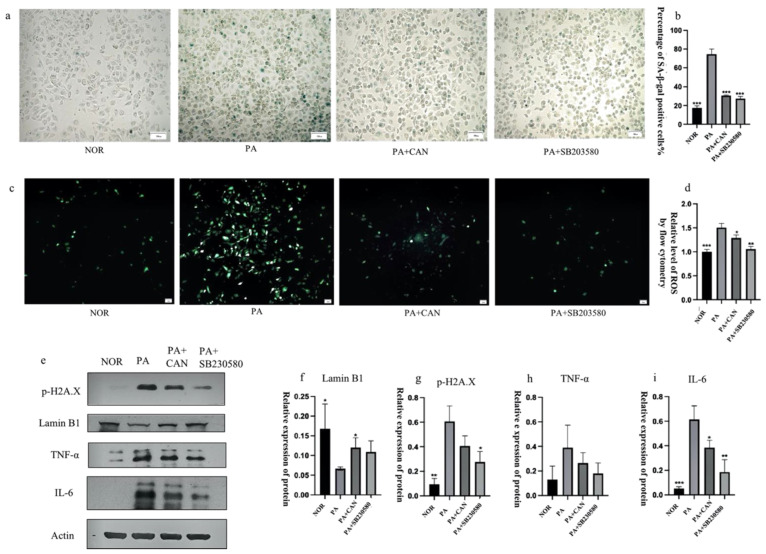
Inhibition of p38 protein activation delayed the senescence of HUVECs. (**a**,**b**) Both CAN (5 μg/mL) and SB230580 (10 μM) reduced the number of β-galactosidase staining-positive cells. (**c**) Both CAN (5 μg/mL) and SB230580 (10 μM) reduced senescence-induced intracellular ROS elevation. (**d**) Results of ROS quantification using flow cytometry. (**e**–**i**) Both CAN (5 μg/mL) and SB230580 (10 μM) reduced the expression of the senescence-related protein p-H2A.X and increased the expression of lamin B1, TNF-α and IL-6 in the SASP. Data are expressed as the means ± SD (*n* = 3); * *p* < 0.05, ** *p* < 0.01, *** *p* < 0.001 vs. PA. All images are magnified to 100×.

The use of SB230580 resulted in a significant decrease in the number of β-galactosidase staining-positive cells (Figure 7a,b), a significant decrease in ROS content (Figure 7c,d), significant decreases in the levels of the senescence signature protein p-H2A.X and SASP inflammatory factors IL-6 and TNF-α and upregulation of lamin B1 protein levels (Figure 7e–i). 

### 3.6. CAN Alleviates PA-Induced HUVEC Replicative Senescence by Inhibiting JNK Activation

**Figure 8:**
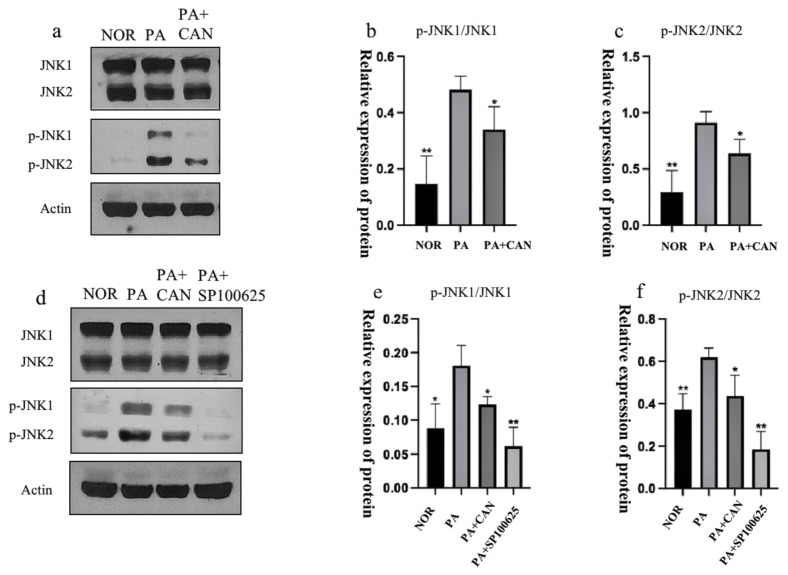
CAN inhibited JNK protein activation. (**a**–**c**) CAN (5 μg/mL) reduced the phosphorylation levels of JNK1 and JNK2 proteins. (**d**–**f**) Effects of JNK inhibitor SP100625 (10 μM) on JNK and JNK2. Data are expressed as the means ± SD (*n* = 3); * *p* < 0.05, ** *p* < 0.01 vs. PA.

JNK is involved in the regulation of multiple cellular responses, including transcription, survival and apoptosis in response to different stimuli [39]. Studies have revealed the function of the JNK pathway in regulating liver homeostasis during aging [40]. The activation of JNK leads to a process of loss or decline in cellular functions that occurs with aging, and brain aging is also importantly linked to JNK activation [41]. We examined the activation of JNK proteins in cells and confirmed that the number of phosphorylated JNK proteins was significantly increased in the PA group (Figure 8a–c). 

**Figure 9:**
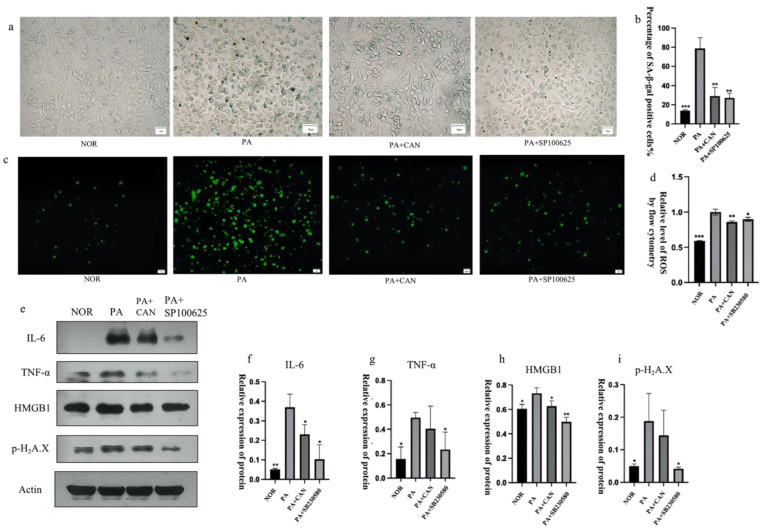
Inhibition of JNK activation alleviated HUVEC cell senescence. (**a**,**b**) Both CAN (5 μg/mL) and SP100625 (10 μM) reduced the number of β-galactosidase staining-positive cells. (**c**) Both CAN (5 μg/mL) and SP100625 (10 μM) reduced senescence-induced intracellular ROS elevation. (**d**) Results of ROS quantification using flow cytometry. (**e**–**i**) Both CAN (5 μg/mL) and SP100625 (10 μM) reduced the SASP expression levels of senescence-associated proteins p-H2A.X, TNF-α, IL-6 and HMGB1. Data are expressed as the means ± SD (*n* = 3); * *p* < 0.05, ** *p* < 0.01, *** *p* < 0.001 vs. PA. All images are magnified to 100×.

Treatment of cells with CAN and the JNK inhibitor SP100625 (Figure 8d–f) revealed a decrease in the number of intracellular β-galactosidase staining-positive cells, a decrease in intracellular ROS levels, and a significant decrease in the senescence signature protein (p-H2A.X) and SASP protein (IL-6, TNF-α, HMGB1) levels compared to the PA group (Figure 9).

### 3.7. CAN Delays PA-Induced Senescence in HUVEC Cells through p38/JNK Dual Targeting

**Figure 10:**
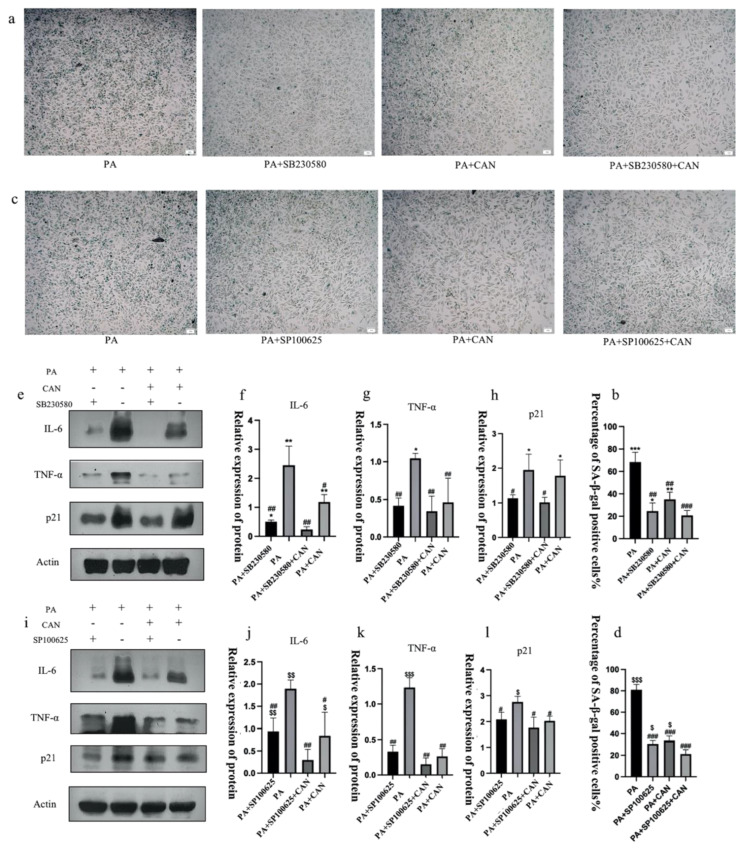
CAN delays PA-induced HUVEC cell senescence by inhibiting p38/JNK activation. (**a**,**b**) The combination of SB230580 (10 μM) and CAN (5 μg/mL) further reduced the number of β-galactosidase staining-positive cells. (**c**,**d**) The combination of SP100625 (10 μM) and CAN (5 μg/mL) further reduced the number of β-galactosidase staining-positive cells. (**e**–**h**) The combination of SB230580 (10 μM) and CAN (5 μg/mL) further reduced SASP factors IL-6, TNF-α and p21. (**i**–**l**) SP100625 (10 μM) in combination with CAN (5 μg/mL) further reduced SASP factors IL-6, TNF-α and p21. Data are expressed as the means ± SD (*n* = 3); * *p* < 0.05, ** *p* < 0.01, *** *p* < 0.001 vs. PA + SB + CAN; $ *p* < 0.05, $$ *p* < 0.01, $$$ *p* < 0.001 vs. PA + SP + CAN; # *p* < 0.05, ## *p* < 0.01, ### *p* < 0.001 vs. PA. All images are magnified to 100×.

To further confirm the ability of CAN to delay cellular senescence, we combined it with the p38 inhibitor SB230580 and JNK inhibitor SP100625. The results showed that the combination of the inhibitors with CAN (PA + CAN + SB group and PA + CAN + SP group) caused further reductions in the β-galactosidase staining-positive rate, decreased SASP inflammatory factor secretion and delayed the senescence of HUVEC cells compared to the PA + CAN group, PA + SB group and PA + SP group (Figure 10). Thus, CAN does not act as a single target but through both the p38MAPK and JNK targets to delay senescence. The mammalian family of MAPKs includes extracellular signal-regulated kinase, p38, and JNK [42]. These results are also consistent with the predicted outcomes from bioinformatics analysis: the MAPK family of p38 and JNK proteins play critical regulatory roles in HUVEC senescence, and CAN and ROS influence the activation of these two targets. 

### 3.8. ROS Inhibitors Attenuate the Activation of p38 and JNK Proteins

**Figure 11:**
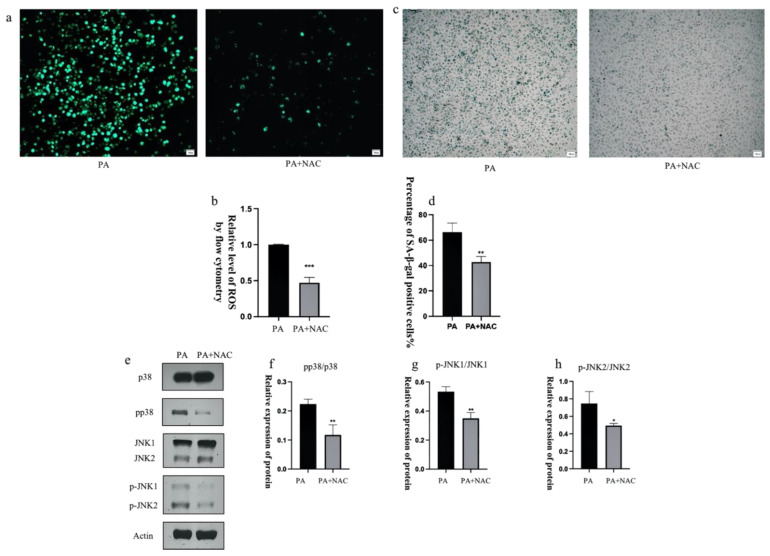
The ROS inhibitor NAC attenuated the activation of p38 and JNK proteins. (**a**) NAC (1 mM) decreased the elevated ROS levels induced by PA. (**b**) Results of ROS quantification using flow cytometry. (**c**,**d**) NAC (1 mM) decreased the number of β-galactosidase staining-positive cells. (**e**–**h**) NAC (1 mM) decreased p38 and JNK phosphorylation levels. Data are expressed as the means ± SD (*n* = 3); * *p* < 0.05, ** *p* < 0.01, *** *p* < 0.001 vs. PA. All images are magnified to 100×.

ROS act as regulators of intercellular signaling and are involved in the regulation of cell growth, development, inflammation and apoptosis [43]. Previous results have demonstrated that intracellular ROS levels appear to be abnormally elevated during senescence, but ROS can also interact with many key signaling molecules [44]. Moreover, elevated ROS levels play a crucial role in the induction and maintenance of cellular senescence [29]. Therefore, we explored the relationship between ROS and p38/JNK signaling molecules. N-acetylcysteine (NAC) is a ROS scavenger and, to investigate the effect of ROS on p38/JNK signaling molecules, we used NAC to scavenge ROS and observed whether ROS reduction could affect the activation of p38/JNK signaling molecules to further identify the targets of drug action. The treatment of senescent cells with the ROS scavenger NAC resulted in a significant reduction in the phosphorylation levels of intracellularly activated p38 and JNK, as well as a significant decrease in the number of β-galactosidase staining-positive cells (Figure 11). These findings suggest that ROS play a pro-senescence role in the PA-induced senescence of PA-treated HUVEC cells, likely mediated by p38/JNK signaling pathways, and that CAN might have a delaying effect on senescence by scavenging ROS in addition to acting directly on p38/JNK signaling molecules. However, further validation should be undertaken in the future.

## 4. Discussion

The therapeutic scope of CAN is no longer limited to lowering blood glucose, and numerous studies have demonstrated the great therapeutic potential of CAN for cardiovascular diseases. In our past study, CAN attenuated cardiomyocyte lipotoxicity through inhibition of the mTOR/HIF-1α pathway, an outcome that, in turn, resulted in the exertion of a protective effect on the diabetic heart [45]. Recent investigations have verified that lipotoxicity induced by hyperlipidemia or increased blood fatty acid levels can increase fat accumulation and promote vascular aging [46,47]. Aging blood vessels are associated with various cardiovascular diseases. Prior work has also found that CAN extended the lifespan of male mice [20,48,49] and delayed age-related lesions in the heart, kidney, liver, and adrenal glands of genetically heterogeneous male mice [49]. These findings reveal the potential role of CAN in delaying aging. However, that possible role has not been conclusively established, and the specific mechanisms by which CAN acts require further investigation. The inference can thus be made that CAN might attenuate vascular aging induced by fat accumulation and exert a protective effect.

In this study, we explored whether CAN could delay vascular senescence and minimize the incidence of cardiovascular disease. CAN could indeed reduce the rate of β-galactosidase staining-positive cells in PA-treated HUVECs and lower the production of excess ROS in PA-treated cells, thereby initially confirming that CAN delays vascular cell senescence. To further investigate the aspects of CAN that delay aging, we examined the effects of CAN on inflammatory factors and DNA damage markers in cells. We confirmed that CAN significantly reduced the inflammatory factors IL-6 and TNF-α in the SASP, and these results were consistent with a previous laboratory study in which we reported that CAN has anti-inflammatory activity and can lower intracellular inflammatory factor levels [13]. CAN was also able to decrease the pro-inflammatory factors HMGB1 and MMP3 in the SASP, as well as the DNA damage marker protein p-H2A.X, and upregulated nuclear fibrillar protein lamin B1. These results suggest that CAN is able to delay cellular senescence by reducing the secretion of the SASP and preventing DNA damage. We also established that CAN is capable of affecting the cell cycle and thus slowing down cellular senescence.

Sustained elevated expression of p38MAPK drives endothelial progenitor cells out of the cell cycle, thereby reducing the number of stem cells and causing tissue senescence [26]. Age-related increases in the levels of endogenous oxidative stress and chronic inflammation activate the p38 MAPK pathway to further accelerate the aging process and the appearance of atherosclerosis-related pathological features in aging tissues. These results are consistent with our findings that the p38MAPK pathway is activated in senescent HUVECs, leading to an elevated cell cycle inhibiting protein expression and elevated SASP, but CAN is able to interact with p38 to reverse the effects from p38 activation. Moreover, evolutionarily conserved JNK signaling is an important genetic determinant of lifespan control [50], and JNK can mediate glycolytic activation leading to aging spermatogonial stem cells [51]. Similar findings were obtained in our study, where empagliflozin, a similar SGLT2 inhibitor to CAN, was able to act on JNK to block its signaling [52].

**Figure 12:**
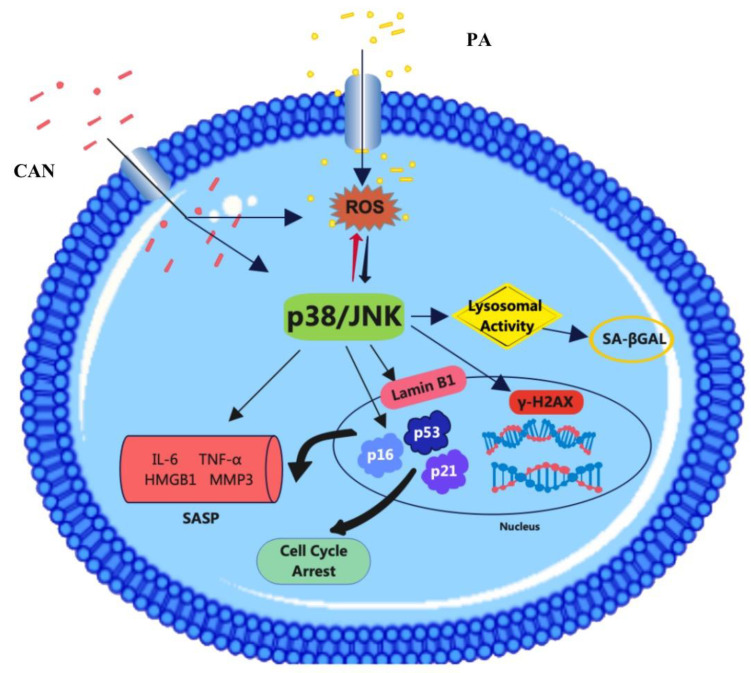
CAN inhibits PA-induced replicative cellular senescence via the ROS/p38/JNK pathway.

Increased fatty acid uptake may promote mitochondrial metabolism and upregulate ROS levels. CAN inhibits mitochondrial metabolism and attenuates the production of ROS, as described previously. Mitochondrial production of ROS is associated with a stress-induced senescence phenotype, and ROS promotes senescence through the p38 MAPK/JNK pathway, thereby regulating downstream targets of senescence and leading to senescence phenotype generation [53,54]. Our findings are in agreement with the results of prior research and indicated that PA can directly cause an abnormal increase in intracellular ROS and then act as a regulatory hub that activates the p38/JNK signaling pathway. We also posit that the effects of CAN might be associated with reduced ROS and/or attenuated p38/JNK signaling in PA-treated cells (Figure 12). However, the exact molecular mechanisms remain to be further investigated.

## 5. Conclusions

In conclusion, our study demonstrates for the first time a specific mechanism of action for CAN in delaying aging through an in vitro model, thereby providing a theoretical basis for the use of CAN as a life-extending drug. Our work identifies the non-hypoglycemic targets of the SGLT2 inhibitor CAN, p38 MAPK and JNK and suggests a theoretical basis for utilizing the ROS/p38/JNK pathway to regulate cellular lifespan. Finally, this research gives a new medicinal value to the SGLT2 inhibitor and offers new therapeutic ideas for lifespan extension.

## Data Availability

The data presented in this study are available in the article and Supplementary Materials.

## Supplementary Materials

The following are available online at https://www.mdpi.com/article/10.3390/antiox12040838/s1, Figure S1: The effects of different concentrations of CAN on HUVEC cell viability were detected using an MTT assay.
