# Supplementary material for: Canagliflozin Delays Aging of HUVECs Induced by Palmitic Acid via the ROS/p38/JNK Pathway
Source: Antioxidants (Basel). 2023 Mar 30;12(4):838. doi: 10.3390/antiox12040838 (PMC10135379; doi:10.3390/antiox12040838)
Supplement: Supplementary file 1 [file antioxidants-12-00838-s001.zip › antioxidants-2258424-supplementary.pdf]

Supplementary Material

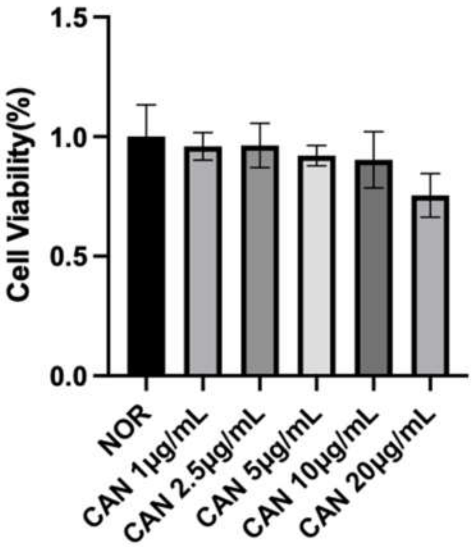

Figure S1. Effect of different concentrations of CAN on HUVEC cell viability was detected using MTT assay. Data are expressed as the mean ± SD (*n* = 3).
